# Adaptive Control for a Two-Axis Semi-Strapdown Stabilized Platform Based on Disturbance Transformation and LWOA-PID

**DOI:** 10.3390/s24165198

**Published:** 2024-08-11

**Authors:** Qixuan Huang, Jiaxing Zhou, Xiang Chen, Qing Li, Runjing Chen

**Affiliations:** 1 School of Electrical Engineering and Automation, Xiamen University of Technology, Xiamen 361024, China; 2222031375@stu.xmut.edu.cn; 2 Xiamen Key Laboratory of Frontier Electric Power Equipment and Intelligent Control, Xiamen 361024, China; 3 Shanghai Institute of Satellite Engineering, Shanghai 201109, China; chenxiang@509.sast.casc; 4 School of Aerospace Engineering, Tsinghua University, Beijing 100085, China; liqing029@mail.tsinghua.edu.cn; 5 School of Computer and Information Engineering, Xiamen University of Technology, Xiamen 361024, China; chenrj@xmut.edu.cn

**Keywords:** disturbance transformation, two-axis semi-strapdown system, LWOA-PID

## Abstract

A two-axis semi-strapdown stabilized platform is a device designed to eliminate aircraft disturbances and ensure the stability of the sensor’s orientation. A traditional two-axis semi-strapdown stabilization platform for aircraft can effectively control disturbance in pitch and yaw channel, but it cannot achieve ideal disturbance control in the roll channel. In order to solve this problem, an adaptive control method based on disturbance transformation and LWOA-PID is proposed. Disturbance transformation is the process of integrating the angular position disturbance of the roll from the previous moment into the combined disturbance of the pitch and yaw at the current moment. This is followed by decoupling the combined disturbance of the pitch and yaw at the current moment, thereby eliminating the disturbance caused by the roll from the previous moment. This process is repeated to achieve the goal of eliminating roll channel disturbances. To ensure the line of sight (LOS) pointing accuracy stability in the two-axis semi-strapdown stabilized platform system for aircraft, a whale optimization adaptive proportional–integral–derivative (LWOA-PID) controller based on Latin hypercube sampling is designed. It is then compared with the classical PID controller in Matlab/Simulink. The simulation results indicate that the disturbance conversion module proposed in this paper can eliminate the impact of roll axis disturbances on the LOS pointing accuracy of the two-axis semi-strapdown stabilized platform for aircraft. Compared to the classical PID controller, the LWOA-PID controller reduces tracking errors for step and sinusoidal signals by 50% and 75%, respectively. It also shortens optimization time by 37.5% compared to the WOA-PID while maintaining the same level of accuracy. Furthermore, when combined with the conversion module, the tracking error is reduced by an additional order of magnitude.

## 1. Introduction

Aerial remote sensing stabilization platforms are devices designed to enhance the quality of images and the accuracy of data acquired by sensors [1]. The two-axis semi-strapdown stabilized platform, due to its small size, light weight, and low cost, holds significant application value in aerial remote sensing stabilization platforms [2]. However, the key to achieving the function of a two-axis semi-strapdown stabilized platform for aircraft lies in whether it can quickly isolate disturbances and ensure the pointing stability of the sensors.

The attitude disturbance of the carrier is the primary factor that affects the tracking performance of the sensors during operation [3]. The aircraft’s attitude inevitably changes during flight, potentially generating corresponding disturbances in angular velocity. If the angular velocity disturbance cannot be compensated for or controlled in a timely manner, it will be transmitted within the stabilized platform, impacting the accurate alignment of the sensor’s LOS axis, thereby ultimately affecting tracking performance. The mechanical structure of the two-axis semi-strapdown stabilization platform for aircraft allows for ideal control only over the roll and pitch channels, making it a coupled underactuated nonlinear system. This type of nonlinear system inherently exhibits chaotic behavior [4]. In [5], it was demonstrated that controllers can effectively manage nonlinear chaotic systems, while in [6], adaptive control for these systems was further explored. Based on controller principles, many academics have conducted significant research on the control strategy of the stabilized platform for aircraft to ensure the pointing stability of the LOS axis of the two-axis semi-strapdown stabilized platform sensor.

A method that combines active disturbance suppression with adaptive fuzzy sliding mode control, which can improve the target tracking capability of stabilized platforms, was proposed by [7]. A multivariable binary adaptive model based on output feedback for a two-axis stabilized platform was proposed by [8] and applied as an actuator on the stabilized platform. A composite control strategy that combines active disturbance compensation and variable gain function techniques to address constraints and disturbances in the output of a visual tracking system for an inertial stabilized platform was proposed by [9]. A sliding mode variable structure control strategy based on the disturbances experienced by the optoelectronic stabilized platform during motion was proposed by [10], significantly reducing the stabilization error of the platform’s velocity loop. The above studies did not analyze the disturbances in stabilization platforms; they merely used controllers to eliminate or compensate for these disturbances to achieve platform stability. To address this issue, this paper conducts an in-depth study of the composition of disturbances in stabilization platforms, categorizing them into aircraft body disturbances and platform-specific disturbances. For aircraft body disturbances, a disturbance conversion module is designed for elimination, while platform-specific disturbances are stabilized using a controller.

Classical PID controllers offer the following advantages: a simple structure, not relying solely on mathematical models, and ease of implementation in engineering. Therefore, classical PID controllers hold a dominant position in various engineering applications, including aerospace, power electronics, and machining. However, when the internal parameters of the system are perturbed and the external environment changes, the previously adjusted controller often exhibits poor adaptability [11].

To enhance the adaptability of classical PID controllers, a fuzzy gain scheduling PID controller for the stabilization of a UAV position and altitude was presented by [12], and its adaptability was validated in MATLAB. A composite control scheme that combines PID and adaptive control was proposed in [13]. The adaptive control provides an adaptive feedforward control signal, while the PID offers feedback control to counteract parametric and nonparametric modeling errors. To improve the line-of-sight stabilization accuracy of the stabilized platform, an adaptive fuzzy controller was designed in [14]. This controller uses a threshold switching method: the adaptive fuzzy controller is applied when the error exceeds the threshold, and PID control is used when the error is below the threshold. Researchers have also conducted studies on PID parameter tuning, including intelligent PID parameter tuning [15,16], PID parameter tuning in multivariable systems [17], nonlinear PID parameter tuning [18,19], and so on. The above studies can be summarized as enhancing the adaptability of PID controllers through real-time adjustment of PID control parameters. It was demonstrated by [20] that the control performance of PID systems with adjustable parameters exceeds that of fixed-parameter PID systems. Based on the concept of adjustable PID parameters, this paper proposes an LWOA-PID controller with the objective of real-time adjustment of PID parameters to enhance the controller’s adaptability.

In this paper, a disturbance transformation method based on delay compensation and an adaptive control approach using LWOA-PID are proposed to ensure the stability and pointing accuracy of the LOS axis for a two-axis semi-strapdown stabilized platform for aircraft. The remainder of this paper is organized as follows: Section 2 deduces the transmission mechanism of angular velocity disturbance in the platform and the principle of LOS axis stability. Section 3 presents the disturbance transformation method and LWOA-PID, and Section 4 conducts simulation verification. Finally, the study is summarized in Section 5.

This paper is dedicated to achieving stable tracking capability for the LOS axis of a two-axis semi-strapdown stabilized platform system used in aircraft, and its main contribution lies in the following:

(1) A disturbance transformation method based on delay compensation is proposed to convert roll channel disturbances into yaw and pitch channel disturbances, thereby achieving effective control of roll channel disturbances in LOS axis of traditional aircraft two-axis semi-strapdown stabilization platforms.

(2) An adaptive control method using LWOA-PID is proposed to ensure the stability of target tracking in the LOS axis of a two-axis semi-strapdown stabilized platform system for aircraft. Compared to the classical PID controller, LWOA-PID offers better tracking ability. Additionally, compared to the traditional WOA-PID, LWOA-PID demonstrates faster optimization speed.

## 2. Coordinate System Definition and Model Analysis

**Figure 1:**
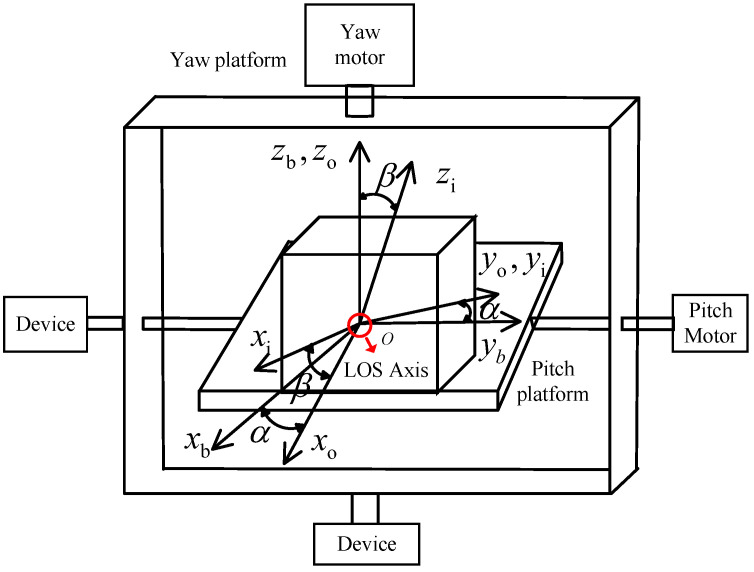
Two-axis semi-strapdown stabilized platform system.

Consider a two-axis semi-strapdown stabilized platform system as depicted in Figure 1. The figure denotes the yaw and pitch platforms. Three reference frames are introduced: an aircraft body-fixed frame (b), a frame (o) fixed to the yaw platform, and a frame (i) fixed to the pitch platform. The center of rotation is in the frame origin, which is assumed to be the same point for the three frames.

$ox_{b}y_{b}z_{b}$ represents the body coordinate system, where $ox_{b}$ is directional in the direction of the aircraft’s head.

$ox_{o}y_{o}z_{o}$ denotes the yaw platform coordinate system, with the angle of rotation around the *z*-axis as α.

$ox_{i}y_{i}z_{i}$ is the pitch platform coordinate system, with the rotation angle around the *x*-axis as β.

The sensors LOS axis coordinate system is denoted by $ox_{g}y_{g}z_{g}$. Since the $ox_{g}y_{g}z_{g}$ is mechanically fixed to $ox_{i}y_{i}z_{i}$, the sensor’s LOS axis coordinate system is equivalent to the pitch platform coordinate system.

These abovementioned coordinate systems have the following transformations:

(1) $$C_{b}^{o}=\left[\begin{array}{ccc} \cos\alpha & \sin\alpha & 0\\ -\sin\alpha & \cos\alpha & 0\\ 0 & 0 & 1 \end{array}\right]$$

(2) $$C_{o}^{i}=\left[\begin{array}{ccc} 1 & 0 & 0\\ 0 & \cos\beta & \sin\beta \\ 0 & -\sin\beta & \cos\beta \end{array}\right]$$

$C_{b}^{o}$ is the transformation from (b) to (o), i.e., if a vector is expressed by its coordinates in frame (b), then $C_{b}^{o}{\vec{v}}_{b}$ gives the coordinates of the same vector in frame (o). Similarly, $C_{o}^{i}$ is the transformation from (o) to (i).

For the angular velocities of frames (b), (o), and (i), the following notations are introduced, respectively:

(3) $$\left\{\begin{array}{c} {\vec{\omega }}_{b}={\left[\begin{array}{ccc} p & q & r \end{array}\right]}^{T}\\ {\vec{\omega }}_{o}={\left[\begin{array}{ccc} \omega_{ox} & \omega_{oy} & \omega_{oz} \end{array}\right]}^{T}\\ {\vec{\omega }}_{i}={\left[\begin{array}{ccc} \omega_{ix} & \omega_{iy} & \omega_{iz} \end{array}\right]}^{T} \end{array}\right.$$

where $\omega_{bx}$, $\omega_{by}$, and $\omega_{bz}$ are the components of the inertial angular velocity of the frame (b) itself, and similarly for the other vectors. We use the notations $\omega_{bx}$, $\omega_{by}$, and $\omega_{bz}$ to represent the pitch, roll, and yaw components, respectively.

According to the definition of Euler angles applied in flight dynamics, Equation (4) is obtained by utilizing Equations (1) and (3).

(4) $${\vec{\omega }}_{o}=\left[\begin{array}{c} \omega_{ox}\\ \omega_{oy}\\ \omega_{oz} \end{array}\right]=\left[\begin{array}{c} \omega_{bx}\cos\alpha +\omega_{by}\sin\alpha \\ \omega_{by}\cos\alpha -\omega_{bx}\sin\alpha \\ \omega_{bz} \end{array}\right]+\left[\begin{array}{c} 0\\ 0\\ \dot{\alpha } \end{array}\right]$$

The vectors are expressed in system (o). ${\left[\begin{array}{ccc} 0 & 0 & \dot{\alpha } \end{array}\right]}^{T}$ represents inertial velocity of the yaw platform. Similarly, between the yaw platform (o) and the pitch platform (i), there exist flight dynamics Euler angles relationships. The following results can be obtained by using Equation (2).

(5) $${\vec{\omega }}_{i}=\left[\begin{array}{c} \omega_{ix}\\ \omega_{iy}\\ \omega_{iz} \end{array}\right]=\left[\left.\begin{array}{c} \omega_{bx}\cos\alpha +\omega_{by}\sin\alpha +\dot{\beta }\\ \cos\beta \left(\omega_{by}\cos\alpha -\omega_{bx}\sin\alpha \right)+\sin\beta \left(\omega_{bz}+\dot{\alpha }\right)\\ -\sin\beta \left(\omega_{by}\cos\alpha -\omega_{bx}\sin\alpha \right)+\cos\beta \left(\omega_{bz}+\dot{\alpha }\right) \end{array}\right]\right.$$

${\left[\begin{array}{ccc} \dot{\beta } & 0 & 0 \end{array}\right]}^{T}$ represents inertial velocity of the pitch platform.

Further, we introduce Equation (5):

(6) $${\vec{\omega }}_{ri}=\left[\begin{array}{c} \omega_{rix}\\ \omega_{riy}\\ \omega_{riz} \end{array}\right]=\left[\begin{array}{c} \omega_{bx}\cos\alpha +\omega_{by}\sin\alpha \\ \cos\beta \left(\omega_{by}\cos\alpha -\omega_{bx}\sin\alpha \right)+\omega_{bz}\sin\beta \\ -\sin\beta \left(\omega_{by}\cos\alpha -\omega_{bx}\sin\alpha \right)+\omega_{bz}\cos\beta \end{array}\right]$$

(7) $${\vec{\omega }}_{di}=\left[\begin{array}{c} \omega_{dix}\\ \omega_{diy}\\ \omega_{diz} \end{array}\right]=\left[\begin{array}{c} \dot{\beta }\\ \dot{\alpha }\sin\beta \\ \dot{\alpha }\cos\beta \end{array}\right]$$

It is apparent that ${\vec{\omega }}_{i}={\vec{\omega }}_{ri}+{\vec{\omega }}_{di}$. The pitch platform angular velocity ${\vec{\omega }}_{i}$, consists of two components: ${\vec{\omega }}_{ri}$, which represents the projection of the aircraft’s angular velocity on the (i) coordinate system, and ${\vec{\omega }}_{di}$, which denotes the angular velocity caused by the motion of the stabilizing platform itself. In order to stabilize the LOS axis, ${\vec{\omega }}_{i}$ should satisfy ${\vec{\omega }}_{i}=0$. However, the aircraft’s two-axis semi-strapdown stabilized platform system lacks a roll axis control motor to directly mitigate roll disturbance, which impacts the stabilization of the sensor’s LOS axis.

**Figure 2:**
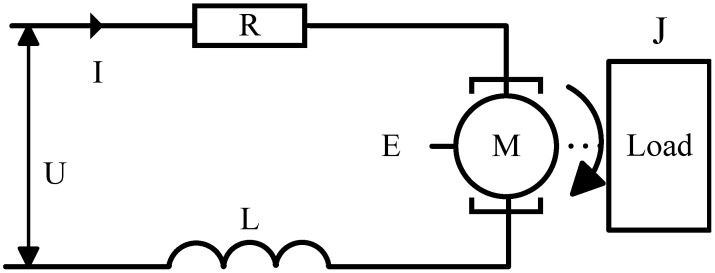
Equivalent circuit diagram of the DC torque motor and platform load.

The semi-strapdown stabilized platform commonly uses a DC torque motor as the control motor. The equivalent circuit diagram of the DC torque motor and platform load is shown in Figure 2:

(8) U=IR+LdIdIdtdt+EE=CeωM=Jω˙+fmωM=CmI

where *U* is the input modulation voltage, *I* is the current between motor windings, *L* is the inductance of the motor windings, *E* is the back electromotive force of the motor, ω is the motor speed, *R* is the motor armature resistance, *J* is the load inertia, $C_{m}$ is the torque constant, and $f_{m}$ is the friction coefficient of the shaft. Typically, the motor current time constant (LLRR) is very small, resulting in minimal impact on system dynamics. Therefore, the motor model can be simplified to

(9) $$\left\{\begin{array}{c} U=IR+C_{e}\omega \\ C_{m}I=J\dot{\omega }+f_{m}\omega \end{array}\right.$$

Applying the Laplace transform to Equation (9), the transfer function between the motor and the platform is obtained as shown in Equation (10).

(10) $$G\left(s\right)=\frac{\omega \left(s\right)}{U\left(s\right)}=\frac{C_{m}}{\left(J+f_{m}\right)Rs+C_{m}C_{e}}$$

## 3. Conversion Module and LWOA-PID Design

From Equation (7), it is evident that when α is a constant, both $\dot{\alpha }$ and $\omega_{diy}$ are equal to zero. In this case, if $\omega_{riy}$ meets the requirement of $\omega_{riy}=0$, then roll axis disturbances can be eliminated. Based on this idea, a conversion module is designed.

### 3.1. Conversion Module

**Figure 3:**
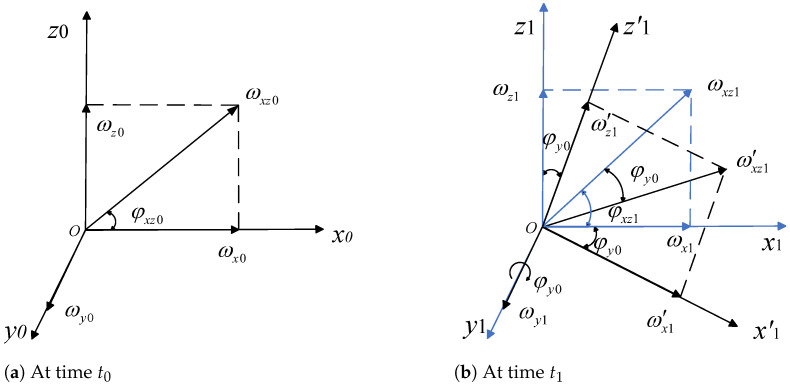
Disturbance relationship.

For the convenience of discussion, $\omega_{rix}$, $\omega_{riy}$, and $\omega_{riz}$ are denoted by $\omega_{x}$, $\omega_{y}$, and $\omega_{z}$. Figure 3a demonstrates the relationship of inertial angular velocity vector components within the (i) coordinate system at time t0. Here, the three-axis disturbances $\omega_{x0}$, $\omega_{y0}$, and $\omega_{z0}$ align with the x0, y0, and z0 axes in the figure, corresponding to pitch, roll, and yaw disturbances, respectively. Additionally, the coordinate system at time t0 is defined as the stable coordinate system.

At the xoz plane, the combined disturbance of $\omega_{x0}$ and $\omega_{z0}$ is represented as $\omega_{xz0}$, with an angle denoted as $\varphi_{xz0}$. At time t0, the disturbance represented by $\omega_{x0}$ and $\omega_{z0}$ is eliminated by their respective motors. However, the absence of a control motor to eliminate $\omega_{y0}$ results in a roll angle $\varphi_{y0}$ at time t1. As a result, the LOS axis coordinate system will rotate around the *y*-axis by the angle $\varphi_{y0}$, leading to instability in the LOS axis.

At time t1, the combined disturbance of $\omega_{x1}$ and $\omega_{z1}$ in the xoz plane is denoted as $\omega_{xz1}$. However, the disturbance of $\omega_{y0}$ at time t0, $\omega_{xz1}$, will next continue to be deflected by an angle $\varphi_{y0}$. The deflected $\omega_{xz1}$ by angle $\varphi_{y0}$ is denoted as $\omega_{xz1}^{\prime }$. It comprises the pitch and yaw disturbance at moment t1, as well as the roll disturbance at moment t0. $\omega_{xz1}^{\prime }$ is decoupled in the stable coordinate system, after which the system is stabilized using pitch and yaw control motors. The disturbance transformation relationship is shown in Figure 3b.

At time t1, compensation for the roll axis disturbance from time t0, followed by compensation at time t2 for the roll axis disturbance from time t1, and subsequently at time t3 for the roll axis disturbance from time t2, continue in this manner. This continuous process achieves eliminating roll channel disturbances. Using this disturbance transformation method, three-axis LOS angular velocity disturbances are converted into two-axis ones. This module is referred to as the conversion module. However, if the deflection angle of the roll axis at time t0 is large, it results in inaccurate feedback, thus impeding the normal work of the conversion module.

The combined disturbance in the oxz plane at time t1 is shown in Equation (11).

(11) $$\omega_{xz\left(t1\right)}=\sqrt{\omega_{z\left(t1\right)}^{2}+\omega_{x\left(t1\right)}^{2}}$$

The angle between the *x*-axis disturbance and the z-axis disturbance at time t1 is shown in Equation (12).

(12) $$\varphi_{xz\left(t1\right)}=\arctan\frac{\omega_{z\left(t1\right)}}{\omega_{x\left(t1\right)}}$$

The equivalent deflection angle in the y-axis direction at time t1 is shown in Equation (13).

(13) $$\varphi_{y\left(t1\right)}=\int_{t0}^{t1}\omega_{y\left(t0\right)}dt$$

The actual deflection angle of the combined disturbance at time t1 is shown in Equation (14).

(14) $$\varphi_{xyz\left(t1\right)}=\varphi_{xz\left(t1\right)}-\varphi_{y\left(t1\right)}$$

The decoupled calculation of the deflection angular velocity at time t1 is shown in Equation (15).

(15) $$\left\{\begin{array}{c} {\omega^{\prime }}_{x\left(t1\right)}=\omega_{xz\left(t1\right)}\sin\left(\varphi_{xyz\left(t1\right)}\right)\\ {\omega^{\prime }}_{z\left(t1\right)}=\omega_{xz\left(t1\right)}\cos\left(\varphi_{xyz\left(t1\right)}\right) \end{array}\right.$$

The algorithm process of the conversion module is shown in Table 1.

### 3.2. LWOA-PID Controller

**Figure 4:**
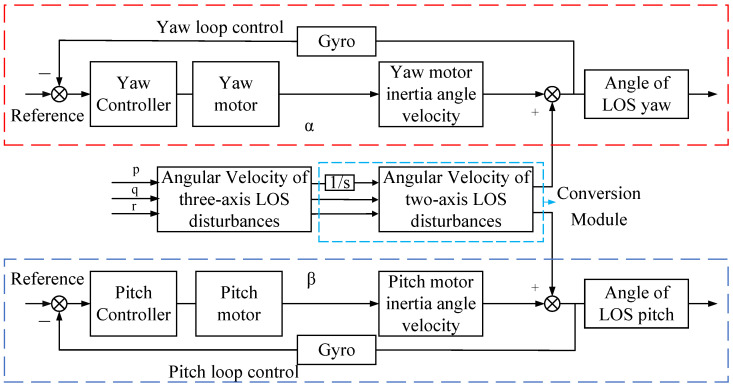
System control diagram.

The two-axis semi-strapdown stabilized platform system for aircraft comprises two loops: the yaw-axis control loop and the pitch-axis control loop. The system control diagram is shown in Figure 4. To guarantee the stability of the two-axis semi-strapdown stabilized platform LOS axis pointing accuracy, an LWOA-PID controller is designed for the yaw/pitch controller module. The LWOA-PID controller enhances the controller’s adaptability by adjusting the PID control parameters in real time.

The whale optimization algorithm simulates the methods that humpback whales use to search for and encircle their prey [21]. This includes three important stages: encircling prey, bubble-net feeding, and searching for prey. Each whale’s position represents a solution, which is continuously updated to eventually obtain the global optimal solution.

Assuming that the current best individual’s position is the optimal foraging location, the other individuals encircle the prey’s position based on this optimal foraging location. The calculation for this process is shown in Equation (16).

(16) $$\left\{\begin{array}{c} D=\left|C\times X^{*}\left(k\right)-X\left(k\right)\right|\\ C=2\times r_{2}\\ X\left(k+1\right)=X^{*}\left(k\right)-A\times D\\ A=2\times a\times r_{1}-a \end{array}\right.$$

In (16), *D* is the distance between the individual and the optimal foraging location; *k* is the current iteration number; $X^{*}\left(k\right)$ is the currently obtained optimal foraging location; $X\left(k\right)$ is the current individual position; *A* and *C* are control coefficients; $r_{1}$ and $r_{2}$ are random numbers in the range [0,1]; *a* is a decay variable that decreases from 2 to 0. The calculation formula is shown in Equation (17).

(17) $$a=2-k\times \frac{2}{k_{sum}}$$

$k_{sum}$ represents the maximum number of iterations. The whale optimization algorithm updates positions by creating a spiral equation to calculate the distance between an individual and the optimal position. The position update calculation formula is shown in Equation (18).

(18) $$\left\{\begin{array}{c} X\left(k+1\right)=X^{*}\left(k\right)+D^{*}\times e^{bl}\times \cos\left(2\pi l\right)\\ D^{*}=\left|X^{*}\left(k\right)-X\left(k\right)\right| \end{array}\right.$$

$D^{*}$ is the distance between the current individual and the current optimal foraging location; *b* is a constant that changes the spiral shape; *l* is a random number in the range [−1, 1]. Contraction encircling and advancing along the spiral path occur simultaneously with equal probability. The calculation formula is shown in Equation (19).

(19) $$X\left(k+1\right)=\left\{\begin{array}{c} X^{*}\left(k\right)-A\times D,p<0.5\\ X^{*}\left(k\right)+D^{*}\times e^{bl}\times \cos\left(2\pi l\right),p>0.5 \end{array}\right.$$

In (19), *p* is a random number in the range [0, 1], which controls the selection of the foraging method.

When |A|>1, peripheral individuals move away from the prey. Unlike the bubble-net feeding stage, which updates positions based on the optimal foraging location, a random individual’s position is chosen as the reference for the next position update. The calculation formula is given as follows:

(20) $$\left\{\begin{array}{c} X\left(k+1\right)=X_{rand}-A\times D\\ D=\left|C\times X_{rand}-X\right| \end{array}\right.$$

In (20), $X_{rand}$ is the position of a random individual. The integral of time-weighted absolute error (ITAE) [22] function is chosen as the fitness function.

(21) $$J=\int_{0}^{\infty }t\left|e\left(t\right)\right|dt$$

Compared to other intelligent optimization algorithms, the WOA is simpler because it only requires setting the population size, number of iterations, and search space range. Depending on the application needs, the population size, number of iterations, and search space range can be modified to customize or adjust the algorithm.

**Figure 5:**
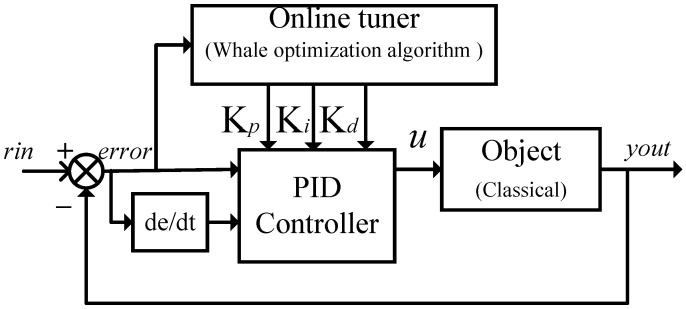
WOA-PID controller diagram.

The general form of a PID controller is as follows:

(22) $$u\left(t\right)=K_{p}e\left(t\right)+K_{i}\int_{0}^{t}e\left(\tau \right)d\tau +K_{d}\frac{de\left(t\right)}{dt}$$

where $u\left(t\right)$ represents the controller output signal, $e\left(t\right)$ denotes the system error, and $K_{p}$, $K_{i}$, and $K_{d}$ are the weighted values of the system error signal and its integral and derivative components, respectively. The classical WOA-PID control diagram is depicted in Figure 5.

The WOA-PID controller achieves system stability by continuously searching for the optimal control parameters using the whale optimization algorithm. The traditional whale optimization algorithm typically uses a uniform random sampling function to initialize the position distribution. However, uniform random sampling can result in an uneven distribution of points in some areas. To address this issue, we propose using a Latin hypercube sampling function to initialize the position distribution in the whale optimization algorithm, aimed at achieving more uniform coverage of the search space.

**Figure 6:**
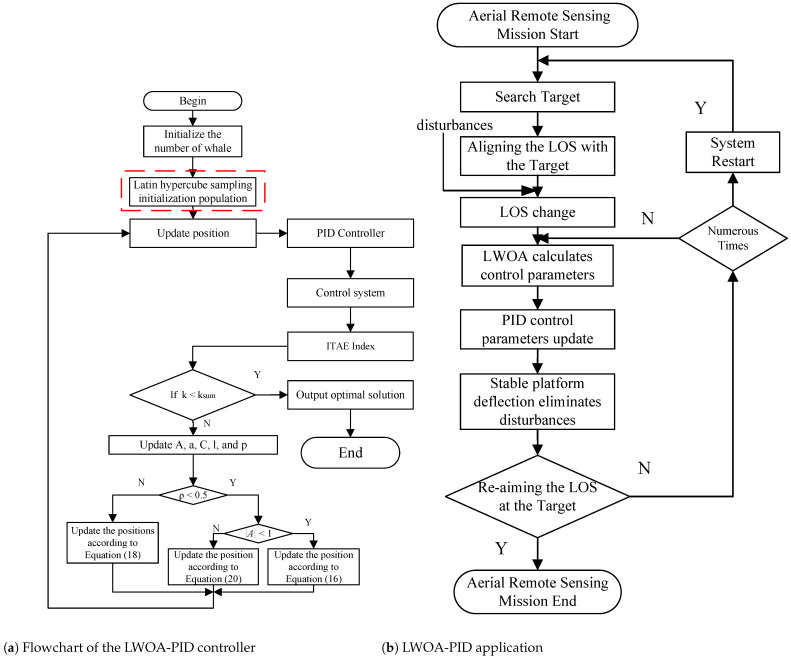
Algorithm procedure and application of LWOA-PID.

The Latin hypercube sampling method was proposed by McKay et al. as a multidimensional stratified sampling technique [23]. It efficiently samples within the variable distribution interval by dividing the interval [0, 1] into N equally spaced, nonoverlapping subintervals and performing independent, equally probable sampling within each subinterval to ensure that sampling points are evenly distributed across the entire distribution interval. Random sampling follows a uniform distribution within the interval [0, 1]. The proposed method, which uses Latin hypercube sampling to initialize the whale optimization positions, is called LWOA-PID. The Latin hypercube sampling algorithm is shown in Table 2. The algorithm procedure and application of the LWOA-PID are shown in Figure 6.

From Figure 6a, the red box highlights the use of Latin hypercube sampling to initialize the whale distribution, as proposed in this paper. The application of the proposed LWOA-PID in aerial remote sensing missions is shown in Figure 6b. The LWOA algorithm primarily consists of three parts: (1) initialization of parameter configurations, (2) initialization of position distribution using Latin hypercube sampling, and (3) global search for the optimal control parameters. The algorithmic process of the LWOA-PID module is shown in Table 3.

The mathematical formula for information entropy in information theory was proposed by Claude Shannon [24]. It is defined as the uncertainty or average amount of information of a probability distribution of a random variable. The formula for calculating entropy is as follows:

(23) $$H\left(x\right)=-\sum_{i=1}^{n}P\left(x_{i}\right)\log_{b}P\left(x_{i}\right)$$

where $H\left(x\right)$ represents the entropy of the random variable *x*, $P\left(x_{i}\right)$ is the probability that the random variable *x* takes on the i-th value, *n* is the number of different possible values that *x* can take, and $\log_{b}$ denotes the logarithm to the base *b*.

A higher entropy value indicates a more uniform distribution of points, covering a larger parameter space, while a lower entropy value suggests that the points are more concentrated in certain areas. The larger the parameter space covered by the optimization algorithm, the better the optimization parameters that can be found. Comparing the entropy values can more intuitively demonstrate the advantages of Latin hypercube sampling.

## 4. Simulink Results and Analysis

In this section, the effectiveness of the proposed conversion module and LWOA-PID adaptive controller is validated using a two-axis semi-strapdown stabilized platform for aircraft as the research object. We assume that the gyroscope feedback is delay-free and error-free. The specifications of the simulation parameters are displayed in Table 4.

### 4.1. Conversion Module Validation

**Figure 7:**
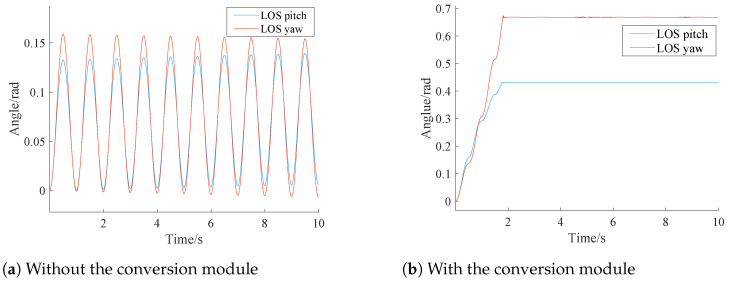
Performance of the conversion module.

The conversion module is designed in Section 3.1. To gain an insight into the performance of the conversion module, simulation associated with the conversion module is conducted and the simulation results are presented in Figure 7.

From Figure 7a, it can be observed that the system itself reduces disturbance amplitudes, but the pitch and yaw angles of the sensor LOS exhibit oscillatory trends.

Figure 7b illustrates that the deflection angles of the pitch and yaw channel tend to stabilize within 2 s. Therefore, the results shown in Figure 7b provide excellent agreement with our statement associated with the conversion module, i.e., the conversion module utilizes lag compensation for roll axis disturbance in continuous time, achieving stabilization of the deflection angle. Nonetheless, the use of the conversion module alone may lead to extra problem like system excessive delay.

### 4.2. Comparison between LWOA-PID and Classical PID

**Figure 8:**
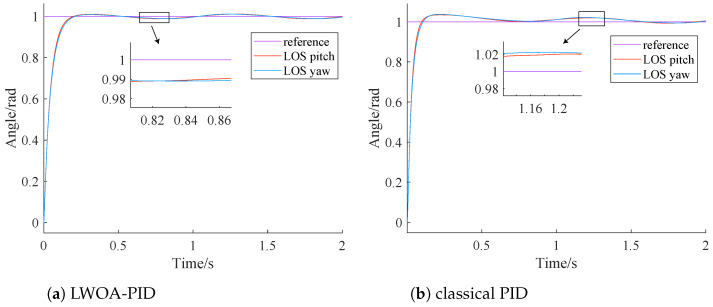
Controller step tracking comparison chart.

**Figure 9:**
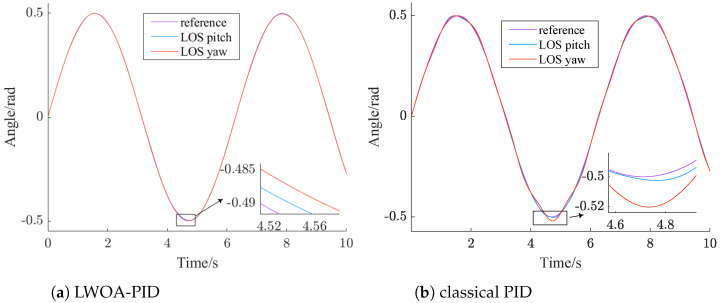
Controller sinusoidal tracking effect comparison.

The LWOA-PID controller is designed according to Section 3.2. The controller parameters of LWOA-PID for tracking step and sinusoidal signals are [99.357, 0, 7.5845] and [100, 100, 14.5286], respectively. According to [25], the parameters of the classical PID controller are [100, 100, 2]. The results of LWOA-PID and classical PID are shown in Figure 8 and Figure 9, and are further summarized in Table 5.

From Figure 8, it can be seen that both the LWOA-PID and the classical PID control methods can stabilize the system within 1 s. The steady-state error of the LWOA-PID controller is 0.01 rad, while the steady-state error of the traditional PID controller is 0.02 rad.

Comparing the PID control parameters of both methods, it is evident that the large initial steady-state error in the classical PID is due to the presence of the integral term in the control parameters. This is because, in the initial stage, there is a significant deviation between the actual value and the desired value. The integration during the phase of large deviation often leads to excessive overshoot in the system.

From Figure 9, it can be seen that the performance of the classical PID controller in tracking a sinusoidal signal is inferior to that of the LWOA-PID controller. The LWOA-PID controller has a maximum tracking error of 0.005 rad, whereas the classical PID controller has a tracking error of 0.02 rad.

Comparing the PID control parameters, it is evident that the classical PID has a larger steady-state error due to the insufficient derivative term. This term, which reflects the rate of change of the error signal, helps introduce an early correction before the error grows too large, thus accelerating system response and reducing adjustment time.

From Table 5, it can be clearly seen that the stable time of the classical PID controller for tracking a step signal is 1.5 times that of the LWOA-PID controller. Additionally, the maximum tracking error of the classical PID controller for a sinusoidal signal is four times that of the LWOA-PID controller. This demonstrates that the LWOA-PID has superior control performance.

The primary reason for this outcome is that the control parameters of the classical PID controller are fixed, whereas the PID controller based on the LWOA optimization algorithm can adjust the PID parameters according to different task requirements to achieve optimal control performance. In [26], genetic optimization algorithms were applied for the self-tuning of PI parameters in permanent magnet synchronous motors (PMSMs) and they demonstrated the feasibility of this optimization algorithm on a 2.3 kW PMSM AC servo platform.

### 4.3. Comparison between LWOA-PID and WOA-PID

**Figure 10:**
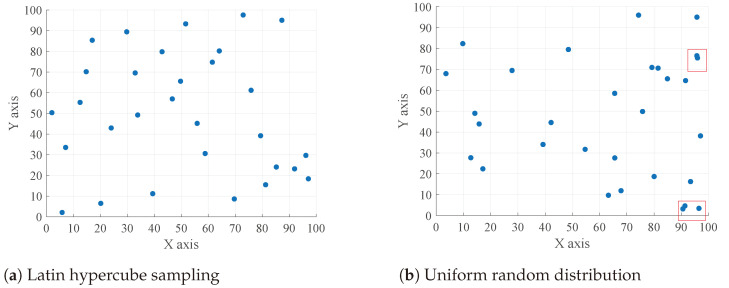
Comparison of two distribution methods.

Latin hypercube sampling can distribute particles more evenly within a specified region, thereby enhancing the global search capability of optimization algorithms and finding the optimal solution with fewer iterations. The comparison between Latin hypercube sampling and uniform random distribution sampling is shown in Figure 10.

**Figure 11:**
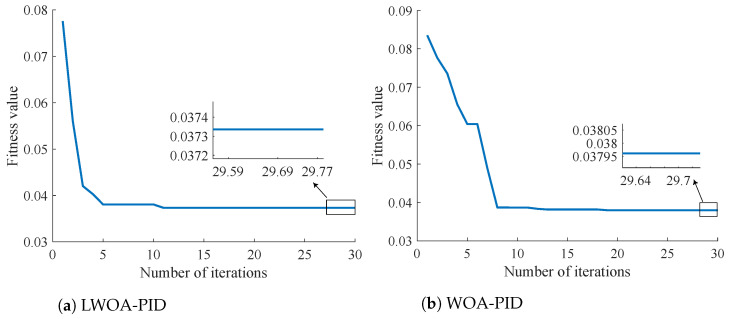
Comparison of LWOA-PID and WOA-PID.

From Figure 10, it can be observed that when using a uniform random distribution sampling function to initialize position distribution, there is a phenomenon where multiple points exist within a specific region, as shown in the red box area in Figure 10b. This decreases the ability to find the optimal solution. Although increasing the number of particles can address this issue, it leads to increased optimization time. In contrast, the Latin hypercube sampling function effectively avoids this problem.According to Equation (23), the entropy values for Latin hypercube sampling and uniform random distribution were calculated as 3.355 and 3.1527, respectively. This further demonstrates that Latin hypercube sampling is superior to uniform random distribution. The comparison between the LWOA-PID and the WOA-PID under the same number of iterations is shown in Figure 11.

From Figure 11, it can be seen that the LWOA-PID algorithm shows a faster optimization speed. By the fifth iteration, the fitness value can reach below 0.04, while the traditional WOA-PID requires eight iterations to achieve the same level. This demonstrates that the LWOA-PID possesses better optimization velocity.

Figure 11 further demonstrates that the more parameter space that is initially covered by the optimization algorithm, the better the results that can be achieved in a shorter time.

### 4.4. Combined Control

**Figure 12:**
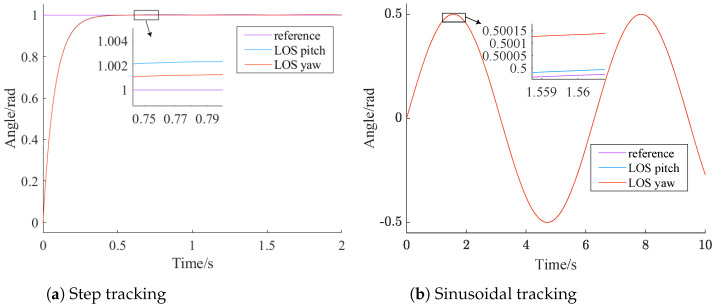
Combined control tracking.

The analyses in Section 4.1 and Section 4.2 indicate that the standalone conversion module can eliminate the impact of roll channel disturbances on the LOS axis, but it has the issue of excessive system delay. Using the LWOA-PID controller alone allows the system to quickly reach a steady state, but it cannot eliminate the impact of roll channel disturbances on the LOS axis. In this section, the conversion module is combined with LWOA-PID and introduced into the system model. Their tracking results for step and sinusoidal signals are shown in Figure 12.

From Figure 12, it can be seen clear that the control method combining the conversion module and LWOA-PID inherits the advantages of both the LWOA-PID controller and the conversion module. Notably, for step signal tracking, the steady-state error was reduced to 0.002 rad, and a steady state was reached within 0.5 s. For sinusoidal signal tracking, the tracking error was reduced by an order of magnitude.

The results in Figure 12 align with the design concept of the conversion module in Section 3. The conversion module aims to achieve $\omega_{riy}=0$, after the LWOA-PID controller, to stabilize the platform by adjusting its angle to counteract pitch and yaw disturbances. According to Equation (7), with no platform deflection, $\omega_{diy}=0$, eliminating the impact of roll disturbances on the LOS. Although the lag compensation characteristic of the conversion module causes minor errors in the LOS, these are negligible.

### 4.5. Sensitivity Analysis of the Conversion Module

**Figure 13:**
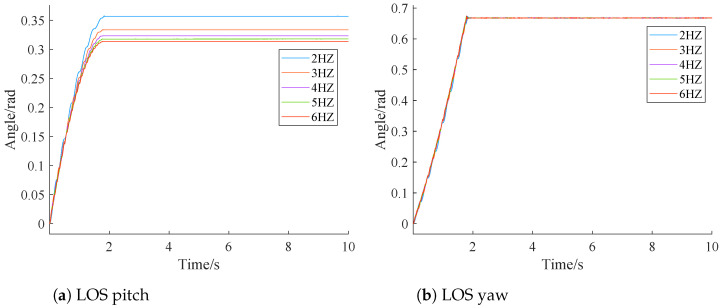
Different frequencies.

**Figure 14:**
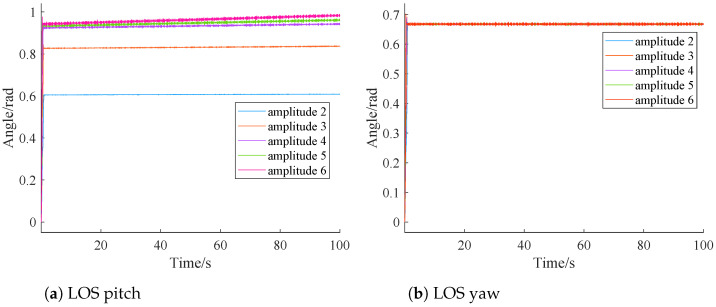
Different amplitudes.

In this section, sensitivity analysis is conducted to explore whether our simulation results are sensitive to frequency and amplitude of input disturbance, respectively. Simulations under different input disturbance amplitude (2, 3, 4, 5, and 6) and under different frequencies (2 Hz, 3 Hz, 4 Hz, 5 Hz, and 6 Hz) are performed. Simulation results are depicted in Figure 13 and Figure 14.

From Figure 13, it is clear that under sinusoidal disturbances of different frequencies, both the pitch and yaw deflection angles of LOS stabilize within 2 s. The yaw deflection angle of LOS does not change with the disturbance frequency, but the stabilized value of the LOS pitch direction decreases with the disturbance.

This is because as the frequency of the disturbance increases, the resistance from the previous moment becomes the control force at the current moment, leading to a decrease in the stabilized deflection angle with higher frequencies.

From Figure 14, it is clear that under sinusoidal disturbances with amplitudes of 2, 3, 4, and 5, both the pitch and yaw deflection angles of LOS tend to stabilize. When the amplitude reaches 6, the steady-state value of LOS yaw remains unchanged, but there is a noticeable increase in the steady-state value of LOS pitch, indicating a decline in the performance of the conversion module.

This is because larger amplitude results in larger roll angles. The principle of the conversion module is similar to lag compensation, where larger disturbances weaken the stability of the feedback loop. This may cause oscillations or overshoot in the system, both of which contribute to an increase in the steady-state value.

Combining Figure 13 and Figure 14, it is clear that while the LOS yaw consistently stabilizes between 0.6 and 0.7 with different disturbances, the LOS pitch does not. This discrepancy is primarily due to the following two reasons:

(1) Reference [27] demonstrated that the pitch axis is more susceptible to disturbances than the yaw axis. This indicates that the pitch axis is more prone to changes when subjected to disturbances.

(2) Larger disturbance amplitudes result in correspondingly larger amplitudes of the disturbance angular velocity. According to Equations (12) to (14), the value of $\varphi_{y}$ increases for the roll channel, while $\varphi_{xz}$ remains unchanged, leading to a change in the range of $\varphi_{xyz}$. The sine function has opposite signs on either side of zero, and the conversion module uses this function to calculate the pitch disturbance after conversion. This introduces errors in the delay compensation of the conversion module.

These two factors together result in a nonunique steady-state value for the LOS pitch. Although the steady-state value of the LOS pitch is nonunique, the conversion module still eliminates the impact of roll channel disturbances on the LOS axis compared to Figure 7a.

## 5. Conclusions

This paper proposed a disturbance transformation method that converts three-axis LOS angular velocity disturbances into two-axis ones, aiming to achieve effective control of roll channel disturbances in traditional two-axis semi-strapdown stabilized platforms for aircraft. We refer to this disturbance transformation method as the conversion module. To enhance the tracking stability of the aircraft’s two-axis semi-strapdown stabilized platform LOS axis, an adaptive control method using LWOA-PID was introduced.

The simulation results indicate that a single conversion modulecan stabilize the disturbance in the roll channel after 2 s. However, it results in excessive delay. Conversely, the LWOA-PID controller method quickly reaches a steady state but fails to stabilize disturbances in the roll channel. The combination of the conversion moduleand LWOA-PID adaptive control not only reduces disturbances in the roll channel on the sensor’s LOS axis but also decreases the time to reach a steady state. The steady-state error is 0.002 rad, with a stabilization time of 0.5 s.

The conversion moduleprovides robust results in the presence of different frequencies in the roll channel, but it performs poorly in controlling disturbance of larger magnitudes. This is an area that requires further study.

## Figures and Tables

**Table 1:** Conversion module algorithm flow.

Conversion Algorithm Table
Input: $\omega_{x\left(t1\right)}$, $\omega_{z\left(t1\right)}$, and $\varphi_{y\left(t1\right)}$
Output: $\omega_{x\left(t1\right)}^{\prime }$ and $\omega_{z\left(t1\right)}^{\prime }$
1. Begin
2. Initialize Conversion Module
3. While (t>0) do:
4. The combined disturbance at the current time is calculated using Equations (11) to (14).
5. Roll disturbances are compensated using Equation (15).
6. Return to Step 4.
7. End while
8. End

**Table 2:** Latin hypercube sampling algorithm.

Latin Hypercube Sampling Algorithm
Input: Population size and dimensionality.
Out: Population randomly distributed in the dimension.
1. Determine the population size N and the population dimension D.
2. Set the interval for variable x as [xmin, xmax], where xmin and
xmax are the lower and upper bounds of the quantity.
3. Divide the interval [xmin, xmax] into N equal subintervals.
4. Randomly select a point within each subinterval in every dimension.
5. Combine the points from each dimension to form the initial population.

**Table 3:** LWOA-PID Module algorithm flow.

LWOA-PID Calculation Process
Input: The number of whale, the values of Ksum and the range of kp, ki, kd
Output: The optimal control parameters
1. Latin hypercube sampling to initialize the whales population
2. Calculate the fitness of each search agent according to Equation (25),
X* = the optimal control parameters
3. while ($k<k_{sum}$)
4. for Update a, A, C, l, and p
5. if1 (p < 0.5)
6. If2 (|A| < 1)
7. Update the position of the current search agent by the Equation (16)
8. Else if2 (|A| >= 1)
9. Update the position of the current search agent by the Equation (20).
10. End if2
11. else if1 (p >= 0.5)
12. Update the position of the current search by the Equation (18).
13. end if1
14. End for
15. Check if any search agent goes beyond the search space and amend it
16. Calculate the fitness of each search agent. Update X* if there is a better solution
17. K = k + 1
18. End while

**Table 4:** The simulation parameter specifications.

Parameter	Value
Input sinusoidal disturbance	1 rad/s, 1 Hz
Number of whales	30
Number of iterate	30
Optimization parameter range	0–100
Initial LOS axis pitch	0 rad
Initial LOS axis yaw	0 rad
$C_{m}$	0.85Nm/A
$C_{e}$	0.85V/rad/s
*J*	$0.0017\;\mathrm{kg}m^{2}$
*R*	4.5Ω
$f_{m}$	0

**Table 5:** Comparison of LWOA-PID and classical PID.

	Optical Yaw		Optical Pitch	
	**Stable Time**	**Max Optical Error**	**Stable Time**	**Max Optical Error**
setp PID	0.82	0.02	075	0.02
setp NPSO-PID	0.54	0.01	0.53	0.01
sinusoidal PID	-	0.02	-	0.002
sinusoidal NPSO-PID	-	0.005	-	0.0025

## Data Availability

Data are contained within the article.
